# Diabetes-linked transcription factor HNF4α regulates metabolism of endogenous methylarginines and β-aminoisobutyric acid by controlling expression of alanine-glyoxylate aminotransferase 2

**DOI:** 10.1038/srep35503

**Published:** 2016-10-18

**Authors:** Dmitry V. Burdin, Alexey A. Kolobov, Chad Brocker, Alexey A. Soshnev, Nikolay Samusik, Anton V. Demyanov, Silke Brilloff, Natalia Jarzebska, Jens Martens-Lobenhoffer, Maren Mieth, Renke Maas, Stefan R. Bornstein, Stefanie M. Bode-Böger, Frank Gonzalez, Norbert Weiss, Roman N. Rodionov

**Affiliations:** 1 Department of Physiology, Saint Petersburg State University, 199034 Saint Petersburg, Russia; 2 Department of Biochemistry, Saint Petersburg State University, 199034 Saint Petersburg, Russia; 3 National Cancer Institute, NIH, Bethesda, MD, 20892, USA; 4 The Rockefeller University, New York, NY, 10065, USA; 5 Stanford University School of Medicine, Stanford, CA, 94305, USA; 6 Institute of Highly Pure Biopreparations, 197110 Saint Petersburg, Russia; 7 University Center for Vascular Medicine, Technische Universität Dresden, 01307 Dresden, Germany; 8 Institute of Clinical Pharmacology, Otto-von-Guericke University, 39120 Magdeburg, Germany; 9 Institute of Experimental and Clinical Pharmacology and Toxicology, Friedrich-Alexander-University Erlangen-Nürnberg (FAU), 91054 Erlangen, Germany; 10 Department of Internal Medicine III, University Hospital Carl Gustav Carus, Technische Universität Dresden, 01307 Dresden, Germany

## Abstract

Elevated levels of circulating asymmetric and symmetric dimethylarginines (ADMA and SDMA) predict and potentially contribute to end organ damage in cardiovascular diseases. Alanine-glyoxylate aminotransferase 2 (AGXT2) regulates systemic levels of ADMA and SDMA, and also of beta-aminoisobutyric acid (BAIB)-a modulator of lipid metabolism. We identified a putative binding site for hepatic nuclear factor 4 α (HNF4α) in *AGXT2* promoter sequence. In a luciferase reporter assay we found a 75% decrease in activity of *Agxt2* core promoter after disruption of the HNF4α binding site. Direct binding of HNF4α to *Agxt2* promoter was confirmed by chromatin immunoprecipitation assay. siRNA-mediated knockdown of *Hnf4a* led to an almost 50% reduction in *Agxt2* mRNA levels in Hepa 1–6 cells. Liver-specific *Hnf4a* knockout mice exhibited a 90% decrease in liver *Agxt2* expression and activity, and elevated plasma levels of ADMA, SDMA and BAIB, compared to wild-type littermates. Thus we identified HNF4α as a major regulator of *Agxt2* expression. Considering a strong association between human *HNF4A* polymorphisms and increased risk of type 2 diabetes our current findings suggest that downregulation of AGXT2 and subsequent impairment in metabolism of dimethylarginines and BAIB caused by HNF4α deficiency might contribute to development of cardiovascular complications in diabetic patients.

Endogenous methylated derivatives of L-arginine, such as asymmetric (ADMA) and symmetric (SDMA) dimethylarginines, have been widely studied as markers and potential mediators of cardiovascular diseases^1^,^2^. ADMA has been proposed to directly cause vascular damage via competitive inhibition and uncoupling of nitric oxide synthases (NOS)^3^. Multiple epidemiological studies reported elevation of plasma ADMA levels in pathological conditions associated with impaired nitric oxide (NO) bioavailability such as atherosclerosis, hypertension and renal failure^4^. In contrast to ADMA, SDMA cannot directly inhibit NOS, but is nevertheless also independently associated with adverse cardiovascular outcomes^5^,^6^. It has been suggested that SDMA contributes to cardiovascular damage by competing with L-arginine for their common transporter^7^,^8^ or by affecting lipid metabolism^9^.

There are two known pathways for enzymatic metabolism of ADMA: hydrolysis to citrulline by dimethylarginine dimethylaminohydrolases 1 and 2 (DDAH1 and DDAH2)^10^, and conversion to asymmetric dimethylguanidinovaleric acid (ADGV) by alanine-glyoxylate aminotransferase 2 (AGXT2)^11^. The first pathway is well characterized, while the second one is still poorly understood. Unlike DDAHs, AGXT2 can metabolize not only ADMA, but also SDMA, which leads to formation of the corresponding α-keto-derivative symmetric dimethylguanovaleric acid (SDGV)^12^. We have demonstrated previously that overexpression of AGXT2 lowers ADMA *in vivo* and protects endothelial cells from ADMA-induced inhibition of NO production^13^. *Agxt2* knockout mice have elevated plasma levels of both ADMA and SDMA and develop hypertension^14^. *AGXT2* polymorphisms have been linked to elevated systemic SDMA levels in humans^15^.

In addition to ADMA and SDMA, AGXT2 can also utilize beta-aminoisobutyric acid (BAIB) as a substrate^16^. *AGXT2* polymorphisms lead to increased BAIB levels in urine (hyper-β-aminoisobutyric aciduria), presumably the most common autosomal recessive metabolic trait in humans^17^ (OMIM 210100). The physiological role of this trait is being currently actively investigated. A recent genome-wide association study revealed a strong association between plasma BAIB levels and serum levels of triglycerides and cholesterol esters^17^. Subsequent experiments in a zebrafish model suggested that BAIB can directly regulate lipid metabolism^17^. Indeed, BAIB was shown to reduce body weight and enhance fatty acid oxidation in mice through increased production of leptin by the white adipose tissue^18^. BAIB has also been proposed to serve as a myokine, which induces browning of white adipose tissue and might be responsible for some of the beneficial effects of exercise^19^.

However, despite the growing evidence that AGXT2 and its substrates may play an important role in the pathogenesis of cardiovascular and metabolic diseases, the mechanisms of regulation of AGXT2 expression and activity are still unknown. We have identified a highly conserved region in the putative promoter sequences of both human and murine *AGXT2*. This region contains a predicted binding site for the transcription factor Hepatocyte Nuclear Factor 4 α (HNF4α). Previous results from chromatin immunoprecipitation using an antibody against HNF4α followed by deep sequencing (ChIP-seq) experiments demonstrated HNF4α binding at this locus in the human hepatocyte cell line HepG2^20^,^21^,^22^. Moreover, Battle and coauthors demonstrated downregulation of *Agxt2* in the liver of *Hnf4a* conditional knockout mice, which suggest an important role of HNF4a in regulation of *AGXT2* expression^23^. The goal of the current study was to test the hypothesis that HNF4α is a major regulator of *AGXT2* expression *in vivo*.

In this study we demonstrated that HNF4α directly binds to the *Agxt2* promoter and serves as a major regulator of *Agxt2* expression *in vitro* and *in vivo*. It is known from previous studies that severe inborn HNF4α deficiency leads to development of maturity-onset diabetes of the young 1 (MODY1)^24^, while mild *HNF4A* polymorphisms are associated with increased risk of type 2 diabetes mellitus and metabolic syndrome^25^,^26^. The current findings therefore suggest an intriguing link between type 2 diabetes and AGXT2-mediated impairment of methylarginine, NO and lipid metabolism.

## Methods

### Plasmids generation

Fragments of the murine *Agxt2* gene promoter region were PCR amplified from C57BL/6J wild type mouse genomic DNA, digested with XhoI and HindIII and cloned into the pGL4.10 vector (Promega). Cloning accuracy was validated by sequencing. Plasmids containing mutations in HNF4α binding site were generated using site-directed mutagenesis. Amplicons were then fused using SOE-PCR technique and cloned into pGL4.10 using XhoI and HindIII restriction sites. All PCR primer sequences are listed in the Supplementary Table S1. Endotoxin-free plasmids for cell line transfections were purified using Qiagen Maxi kit (Qiagen).

### Cell culture

Hepa 1–6 cells (CLS, Germany) were cultured to 70% confluence in Dulbecco’s modified Eagle’s medium/F12 (DMEM/F12) containing 10% fetal bovine serum (FBS) and 1% penicillin-streptomycin. NIH3T3 cells (CLS, Germany) were cultured in Dulbecco’s modified Eagle’s medium (DMEM) containing 10% fetal bovine serum (FBS) and 1% penicillin-streptomycin. For transfections cells were seeded at 6000 cells/well in 96-well plate (Greiner Bio-One) and incubated 24 hours.

### Animals

*Hnf4a*^*F/F*;*AlbERT2cre*^ mice with tamoxifen-inducible liver-specific *Hnf4a* deficiency as well as the control *Hnf4a*^*F/F*^ mice were previously generated and characterized by Dr. Gonzales’ group^27^. Animals were fed a diet containing tamoxifen (1 g/kg diet) for four days and returned to regular chow for additional four days. On the eighth day all animals were euthanized and tissues collected for analysis. Mice were housed in a temperature- and light-controlled facility and given food and water *ad libitum*. All animal studies were performed in accordance with the guidelines and approval of the NCI, National Institutes of Health, Animal Care and Use Committee.

### Plasmid transfection and Dual-Luciferase assay

Master mixes of FuGene transfection reagent (Promega) and reporter plasmids in serum-free Opti-MEM medium (Gibco) were added to the cells. Plasmids based on the pGL4.10 vector (Promega) containing parts of murine *Agxt2* gene putative promoter region and carrying firefly luciferase as a reporter gene were co-transfected with the pGL4.74 vector (Promega) containing *Renilla* luciferase gene with HSV TK-promoter as an internal control for transfection efficiency. Cells were assayed with Dual-Luciferase Assay kit (Promega) according to the instructions of the manufacturer twenty four hours after transfection with reporter plasmids. The signal was acquired using a luminometer with auto-injection system Berthold Centro XS^3^ LB 960. Chemiluminescence intensity variation was then normalized to the *Renilla* luciferase signal.

### siRNA transfection

Lipofectamine RNAiMAX transfection reagent (Invitrogen) and different siRNAs in serum-free Opti-MEM medium (Gibco) diluted 1:1 with water were added to cells. Scrambled oligonucleotides Stealth MediumGC (12935–300, Life Technologies, USA) and Ambion Negative control 1 (AM4611, Life Technologies, USA) were used as negative controls. Cells were incubated for 96 hours without medium change followed by analysis of gene expression.

### RNA extraction, cDNA synthesis and qPCR

RNA from siRNA-treated cells was extracted with Cells-to-Ct kit (Ambion), cDNA was subsequently synthesized with the same kit. RNA extraction from murine tissues was performed using RNeasy Plus Universal kit (Qiagen), cDNA was synthesized with High Capacity RNA Reverse Transcription Kit (Applied Biosystems). qPCR was performed using ABI 7300 machine (Applied Biosystems) with PowerSYBR reagent (Applied Biosystems) in 96-tube plates. Standard reaction conditions were used and all genes were assayed on the same plate. The sequences of the primers for qPCR are listed in the Supplementary Table S1.

### Immunoblotting

After the 96 hr incubation, cells were washed with PBS, scraped off the plates and centrifuged at 500 g for 10 minutes. Cell pellets were lysed with RIPA buffer containing Roche complete protease inhibitor cocktail (Roche), sonicated and frozen at −80 °C. Protein concentrations in samples were determined using the BCA protein assay (Thermo Scientific, USA). Samples (15 μg of protein/lane) were separated by SDS-PAGE under reducing conditions on 10% polyacrylamide gels and transferred to PVDF membranes (Roti-PVDF, Carl Roth, Germany). Membranes were probed with 1 μg/mL mouse monoclonal anti-HNF4a antibodies clone H1415 (Invitrogen, USA) for 1 h at room temperature followed by incubation with 1 μg/mL HRP-conjugated goat-anti-mouse secondary antibodies (Becton Dickinson) for 1 h at room temperature. To control for sample loading, the membranes were re-probed with 1 μg/ml anti-beta-actin HRP-conjugated mouse monoclonal antibodies (Sigma) for 1 hr at room temperature. Bands were visualized using Lumi-Light Western Blotting Substrate (Roche, Switzerland).

Liver and kidneys isolated from three Cre positive and three Cre negative *Hnf4a* Flox/Flox mice were homogenized in RIPA buffer containing Halt Protease Inhibitor Cocktail (Thermo Scientific). 50 μg of protein extract was used for SDS-PAGE and Western blot analysis using Biorad TGX precast gels and Trans-Blot Turbo PVDF transfer packs, respectively. Membranes were incubated with antibodies against HNF4A (H1415, Perseus (Tokyo, JP)) then re-probed with β-actin (ab8227, Abcam) as a loading control.

### Chromatin immunoprecipitation assay

Chromatin was extracted from 20 million Hepa 1.6 or NIH3T3 cells with ChIP-IT Express Enzymatic kit (Active Motif), according to the manufacturer’s recommendations. Every ChIP reaction was performed with 5 μg of mouse anti-HNF4α antibodies (H1415, Life Technologies). Immunoprecipitated DNA and Input DNA were purified with Chromatin IP DNA Purification Kit (Active Motif). It was subsequently analyzed by qPCR to measure the relative enrichment of the fragments of interest in the total input of ChIP DNA fragments. The negative control ChIP primers were designed to amplify fragments downstream of the *Agxt2* predicted transcription start sites. The positive control primers were designed to amplify the region of hepatocyte nuclear factor 1 alpha (*Hnf1a*) promoter, which has been shown previously to comprise an active HNF4A binding site^28^. Murine fibroblast cell line NIH 3T3 was used as an additional negative control. ChIP-IT Control qPCR Kit for Mouse (Active Motif) was used as an additional control.

### Biochemical measurements

Measurements of ADMA and ADGV in tissue lysates for determination of AGXT2 activity were performed using HPLC-MS-MS as previously described^11^,^29^. AGXT2 activity was assessed as a production rate of the AGXT2-specific metabolite of ADMA–asymmetric dymethylguanovaleric acid (ADGV)-per mg of tissue after incubation with isotope-labeled ADMA^30^. Plasma levels of ADMA, SDMA, ADGV, SDGV and BAIB were determined using the corresponding HPLC-MS-MS methods^31^,^32^. Baseline separation was achieved by a HILIC column and identity was confirmed by reference substances and multiple mass transitions. For SDGV, cell-culture-derived isotope labelled [2H6]-SDGV was used as the internal standard, therefore, only relative units (i.e. area ratios) are provided.

### Statistics

All quantitative data are presented as mean ± SEM. Normality of distributions was tested using D’Agostino & Pearson omnibus normality test and Shapiro-Wilk normality test. Statistical comparisons between two groups were performed using Student’s t-test and between multiple groups-with Dunnett’s multiple comparison test. Differences were considered statistically significant at p-value < 0.05. All analyses were performed in Prism 6 (GraphPad Software).

## Results

### HNF4α is a candidate regulator of AGXT2 expression

**Figure 1:**
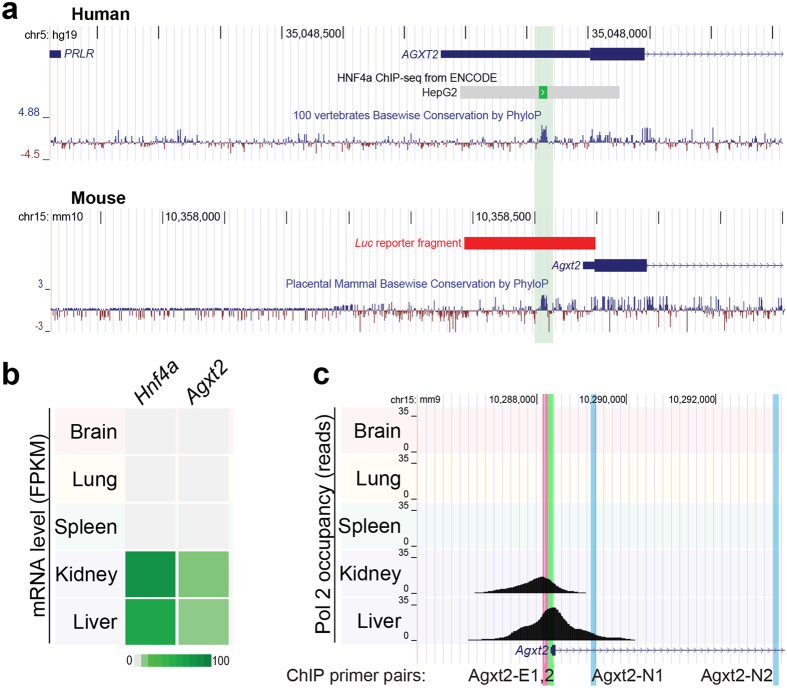
Conserved regulatory element in *Agxt2* promoter corresponds to HNF4A binding site. (**a**) A single conserved element is located in 5′ UTR of human *AGXT2* and upstream of mouse *Agxt2* gene. UCSC Genome Browser view of human (hg19) *AGXT2* and mouse (mm10) *Agxt2* loci, with PhyloP conservation tracks shown below. For human locus, ENCODE results identifying HNF4A binding in HepG2 cell lines are shown. For mouse locus, fragment used in luciferase reporter assay (Fig. 2) is indicated by a red bar. Location of primers used for ChIP (Fig. 3b) is indicated with colored vertical bars. (**b**) mRNA levels of *Hnf4a* and *Agxt2* correlate in mouse tissues according to RNA-seq data. Heat map indicates transcript abundance in fragments per kilobase of exon per million fragments mapped (FPKM)^67^. (**c**) Pol2 occupancy at *Agxt2* promoter is high in the tissues expressing *Hnf4a*. UCSC Genome Browser view of mouse *Agxt2* locus with corresponding sets of Pol2 ChIP-Seq reads (generated by Bing Ren’s lab)^20^. Positions of primers, used for ChIP experiments are marked with colored lines, names are in the bottom.

We performed a conservation analysis of the mammalian sequences upstream of the first exon of *AGXT2* gene using the VISTA software^33^. The only highly conserved region found in mammals within the predicted *AGXT2* core promoter region was located −89 bp to −77 bp upstream relative to the murine *Agxt2* gene translation start site (according to mm 9 genome annotation, *Agxt2* has multiple transcription start sites, therefore all coordinates in this manuscript are reported in relation to the single translation start site). The identified region contained the HNF4α consensus binding sequence^34^. Furthermore, this region overlapped with the HNF4α footprint from the ChIP-seq analysis using anti-HNF4α antibodies performed in HepG2 cells^20^,^21^,^22^ (Fig. 1a).

Analysis of the publicly accessible microarray data obtained from 22 C57BL/6 wild type mouse tissues (Gene Expression Omnibus database, dataset GDS3142^35^,^36^ demonstrated a strong correlation between *Hnf4a* and *Agxt2* expression levels (Pearson’s R = 0.736). Notably, no significant correlation was observed between expression of *Hnf4a* and the two genes flanking *Agxt2* on mouse chromosome 15 (R = 0.02 and −0.14 for upstream *Prlr* and downstream *Dnajc21*, respectively), suggesting that HNF4α-dependent regulation is specific to *Agxt2*. Further, the strongest expression of both *Agxt2* and *Hnf4a* was observed in the liver and the kidney (Fig. 1b). These tissues also showed high RNA polymerase II (Pol II) occupancy at the *Agxt2* promoter in the corresponding sets of ChIP-seq reads^20^, suggesting that HNF4α directly contributes to transcriptional regulation of *Agxt2* (Fig. 1c).

### Disruption of the predicted HNF4α binding site reduces activity of the Agxt2 promoter

**Figure 2:**
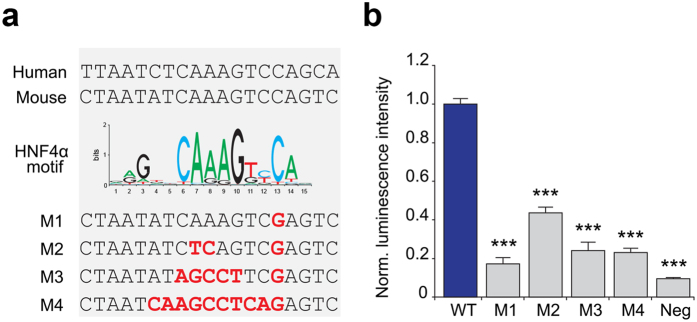
HNF4α binding site is necessary for *Agxt2* proximal promoter function. **(a**) Conserved sequence in the *AGXT2* core promoter region (top) matches the known HNF4α binding site consensus sequence (middle)^68^. Bottom, sequences of disrupted HNF4α binding sites in four mutant constructs (M1–M4), which were tested in the luciferase reporter experiment. Mutated nucleotides are shown in bold red. **(b**) Disruption of the HNF4α recognition sequence in the *Agxt2* core promoter reduced expression of the reporter gene, as demonstrated by the luciferase activity assay in the lysate of the cells expressing the *Agxt2* promoter/luciferase reporter constructs. WT-intact fragment containing *Agxt2* proximal promoter. M1–M4–mutated constructs, containing the mutations shown in the panel (a). Neg-promoterless luciferase construct (negative control). Average of 9 independent biological replicates, error bars indicate SEM, one-way ANOVA, Dunnett’s multiple comparison test: ***P < 0.0001 for all.

We then tested the functional activity of the predicted HNF4α binding site in the murine *Agxt2* promoter using a luciferase reporter assay. The intact construct encoded the predicted core promoter region spanning −211 to +1 of the murine *Agxt2* promoter followed by the firefly luciferase reporter. Four mutant constructs (M1–M4) were generated: the M1 and M2 contained previously described functionally significant SNPs incorporated into the HNF4A binding site^37^,^38^, while in the M3 and M4 the entire consensus sequence was disrupted^34^ (Fig. 2a).

The constructs were expressed in the murine hepatic cell line Hepa 1–6. Firefly luciferase activity measured in the lysates of Hepa 1–6 cells transfected with the mutated constructs was on average 75% lower (maximum 83% in case of the mutated construct 1) than in the lysates of cells transfected with the construct containing the intact promoter (Fig. 2b). The signal of Renilla luciferase used as an internal reference was consistent among the samples.

### HNF4α directly binds to Agxt2 promoter

**Figure 3:**
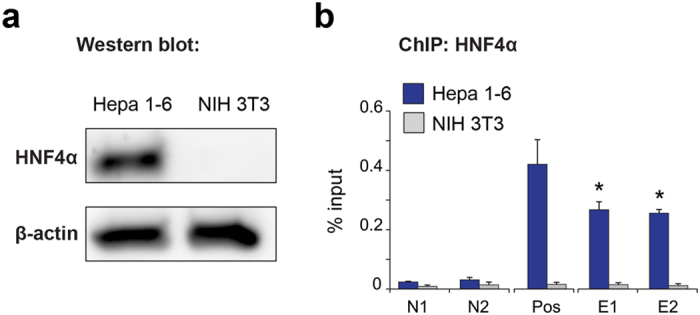
HNF4α directly binds to its consensus site in the Agxt2 promoter. **(a**) Western blotting demonstrates HNF4α protein expression in the murine hepatocyte cell line Hepa 1–6, but not in the murine fibroblast cell line NIH 3T3. **(b**) ChIP analyses of HNF4α binding to DNA sequences of interest. Dark blue–Hepa 1–6 cells, which express both Hnf4a and Agxt2, Gray–NIH 3T3 fibroblasts, which express neither Hnf4a, nor Agxt2 (negative control). N1 and N2 (negative controls) -sequences 1 and 5 kb downstream of Agxt2 promoter, lacking known HNF4A consensus binding site. Pos (positive control) –sequence comprising previously shown HNF4α binding site in Hnf1a gene promoter region. E1 and E2 (experiment) -two different primer pairs, both centered around the predicted HNF4α binding site in the Agxt2 promoter. Average of 3 (for N2 and P) or 4 (for N1, E1 and E2) independent biological replicates, error bars indicate SEM, Mann-Whitney test *P = 0.02866.

We performed a ChIP assay with anti-HNF4α antibodies to determine whether HNF4α can directly bind to the murine *Agxt2* promoter. We chose two cell lines for this experiment: the murine hepatocyte cell line Hepa 1–6, which expresses *Hnf4a*, and the murine fibroblast cell line NIH 3T3, which does not express *Hnf4a* (Fig. 3a).

A qPCR analysis of the chromatin precipitated with anti-HNF4A antibodies from Hepa 1–6 cell lysates showed enrichment of the DNA fragments containing the putative HNF4α binding site in the *Agxt2* promoter, when compared to the negative control regions. The positive control region, containing known HNF4α binding site, also demonstrated enrichment compared to the negative control regions. The negative control experiment performed with the lysate from NIH 3T3 cells demonstrated no enrichment in any of the tested regions (Fig. 3b).

### siRNA-mediated knockdown of Hnf4a reduces Agxt2 expression

**Figure 4:**
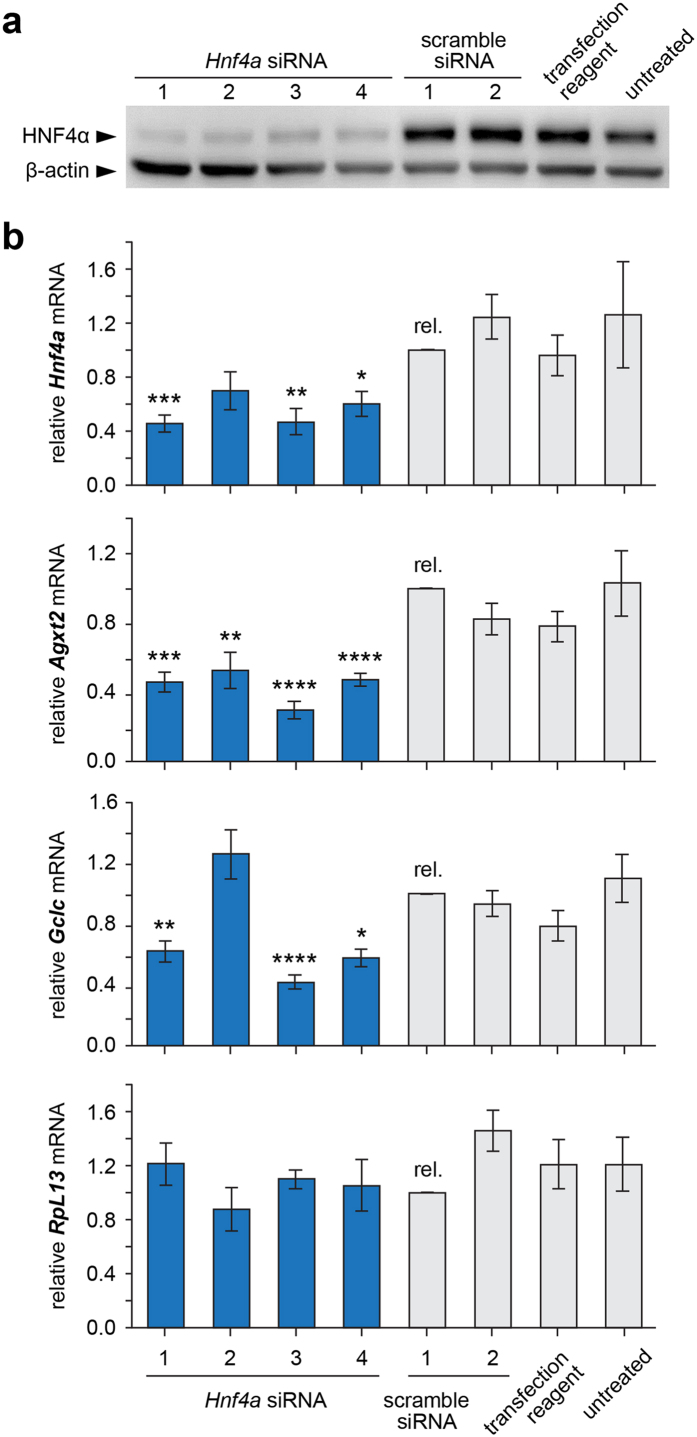
siRNA-mediated knockdown of *Hnf4a* reduces *Agxt2* expression in cultured murine hepatocytes. **(a**) Validation of knock-down efficiency of *Hnf4a* in Hepa 1–6 cell line. Western blotting analysis with anti-HNF4α antibodies in whole cell lysates after treatment with four distinct siRNAs against *Hnf4a* and four negative controls: two scramble siRNAs, transfection reagent alone and untreated cells. Beta-actin is used as loading control. (**b**) Reduced *Agxt2* mRNA accumulation in Hepa 1–6 cells after *Hnf4a* knock-down. qPCR was performed in four siRNA-treated and four negative control cell lines, with ΔΔCt quantification relative to scramble 1 control. *Gclc* serves as positive control. *RpL13* serves as negative control. Average of 9 independent biological replicates, error bars indicate SEM, one-way ANOVA, Dunnett’s multiple comparison test: *P < 0.05; **P < 0.01; ***P < 0.001; ****P < 0.0001. (Precise P-values, *Hnf4a*: *Hnf4a* siRNA 1–0.0001, *Hnf4a* siRNA 2–0.003, *Hnf4a* siRNA 4–0.0137*; Agxt2*: *Hnf4a* siRNA 1–0.0001, *Hnf4a* siRNA 2–0.0089, *Hnf4a* siRNA 3 − <0.0001, *Hnf4a* siRNA 4− <0.0001*; Gclc*: *Hnf4a* siRNA 1–0.0032, *Hnf4a* siRNA 3 − <0.0001, *Hnf4a* siRNA 4–0.0005).

Next we downregulated expression of *Hnf4a* in cultured Hepa 1–6 cells by siRNA-mediated knockdown to determine whether HNF4α is required for endogenous *Agxt2* expression. Four different siRNAs against *Hnf4a* mRNA were used with the following negative controls: two different scrambled siRNAs, the mock control without siRNA and intact cells. Efficiency of *Hnf4a* knockdown was confirmed at the protein level by Western blotting (Fig. 4a). mRNA levels of *Agxt2* and *Hnf4a* were measured by RT-qPCR. Glutamate-cysteine ligase (*Gclc*) mRNA level was assessed as an additional positive control for *Hnf4a* knockdown, since this gene is known to be regulated by HNF4α^35^. 60S ribosomal protein L13 (*RpL13*) mRNA level was assessed as an additional negative control for global effects of *Hnf4a* knockdown, since we did not expect regulation of *RpL13* expression by HNF4α.

Transfection of Hepa 1–6 cells with siRNAs against *Hnf4a* led to a 40% decrease in *Hnf4a* mRNA levels and about 55% decrease in *Agxt2* mRNA levels compared to the control scrambled siRNA. The levels of *Gclc* mRNA were decreased by approximately 40% in the cells expressing anti-HNF4α siRNAs 1, 3 and 4 indicating efficiency of HNF4α knockdown. The levels of *Gclc* mRNA were increased by 19% (n.s.) in the cells expressing anti-HNF4α siRNA 2 likely due to nonspecific activity of this particular *Hnf4a* siRNA (Fig. 4b). No difference in *RpL13* gene expression between the samples was observed (Fig. 4b).

### Agxt2 mRNA levels are decreased in Hnf4a knockout mice

**Figure 5:**
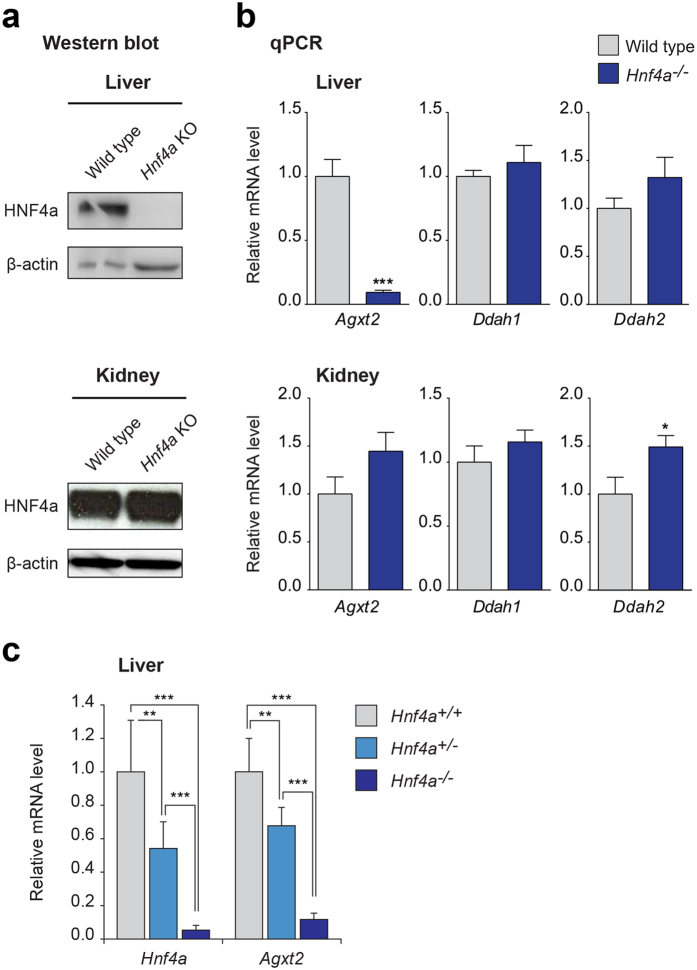
Expression of *Agxt2* in inducible liver-specific *Hnf4a* knockout mice. (**a**) Western blotting of liver and kidney lysates from liver-specific *Hnf4a* knockout mice (*Hnf4a* KO) and the wild type littermates (wild type). Beta-actin was used as loading control. **(b)** *Agxt2*, *Ddah1* and *Ddah2* mRNA levels in the liver and kidneys of liver-specific *Hnf4a* knockout mice and littermates determined by qPCR. n = 7 for WT, n = 6 for *Hnf4a* knockout mice, error bars indicate SEM, Mann-Whitney test: *P = 0.04; ***P < 0.0001. **(c)** *Agxt2* mRNA levels in the liver of homozygous and heterozygous liver-specific *Hnf4a* knockout mice and littermates determined by qPCR. n = 7 for both heterozygous and homozygous *Hnf4a* knockout mice, n = 8 for WT, error bars indicate SEM, one-way ANOVA, Tukey’s multiple comparison test: *Hnf4a* level*: Hnf4a*^+/+^ vs. *Hnf4a*^+/−^, **P = 0.0012; *Hnf4a*^+/+^ vs. *Hnf4a*^−/−^, ***P < 0.0001; *Hnf4a*^+/−^ vs. *Hnf4a*^−/−^, ***P = 0.0009; *Agxt2* level*: Hnf4a*^+/+^ vs. *Hnf4a*^+/−^, **P = 0.0006; *Hnf4a*^+/+^ vs. *Hnf4a*^−/−^, ***P < 0.0001; *Hnf4a*^+/−^ vs. *Hnf4a*^−/−^, ***P < 0.0001.

We took advantage of the previously characterized inducible liver-specific *Hnf4a* knockout mice^27^ to determine whether HNF4α regulates *Agxt2* expression *in vivo.* No HNF4α protein was detected by Western blot in the liver of knockout animals after induction of Cre recombinase expression by tamoxifen. Deletion of *Hnf4a* led to greater than 90% decrease in *Agxt2* mRNA levels in the liver. No significant changes in the *Agxt2* mRNA levels were observed in the kidneys, where HNF4α protein levels remained intact (Fig. 5a). Partial deletion of *Hnf4a* in heterozygous *Hnf4a* knockout mice led to respective decrease in *Agxt2* mRNA levels in a dose-dependent manner (Fig. 5c). Neither the liver, nor the kidneys showed any compensatory changes in *Ddah1* expression. The levels of *Ddah2* remained unchanged in the liver, but were increased by about 50% in the kidney (p < 0.05) (Fig. 5b).

### AGXT2 enzymatic activity is decreased and metabolites levels are changed in plasma of Hnf4a knockout mice

**Figure 6:**
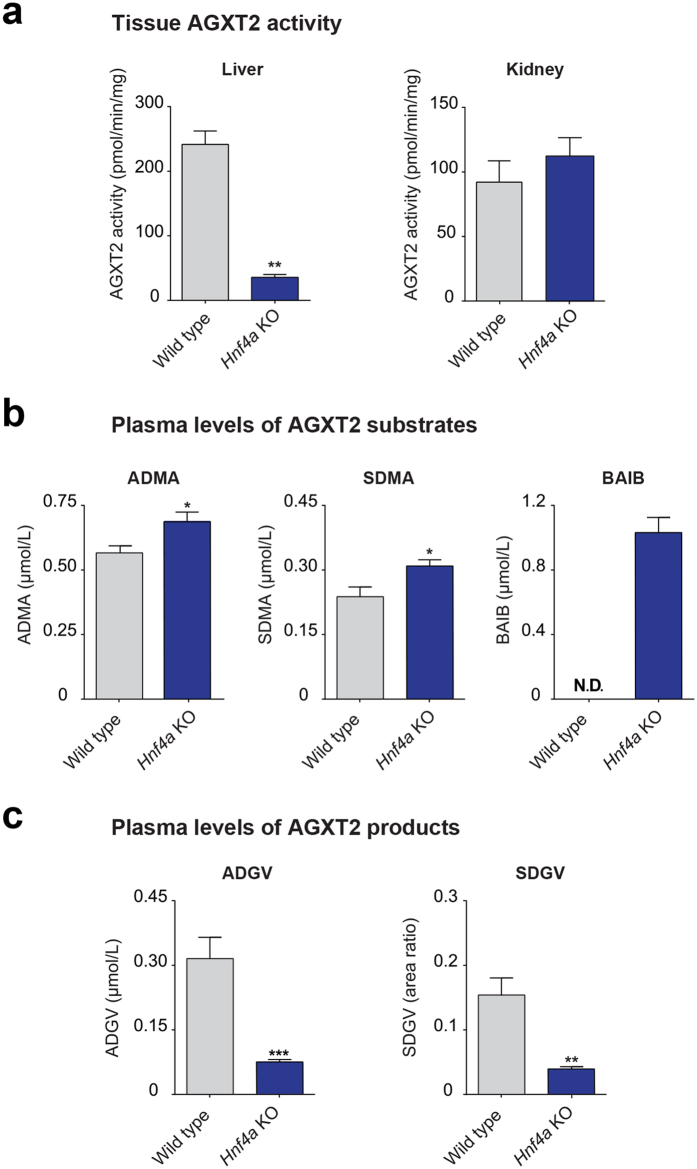
AGXT2 activity and metabolites levels in plasma of inducible liver-specific *Hnf4a* knockout mice. **(a)** AGXT2 activity was assessed as a production rate of AGXT2-specific metabolite of ADMA–asymmetric dymethylguanovaleric acid (ADGV) per mg of tissue after incubation with isotope-labeled ADMA. ADGV concentrations were determined by HPLC-MS-MS. n = 7 for WT, n = 6 for *Hnf4a* knockout mice, error bars indicate SEM, Mann-Whitney test: **P = 0.0012. **(b)** AGXT2 substrates levels in plasma of liver specific *Hnf4a* knockout and wild type mice. ADMA, SDMA, and BAIB concentrations determined by HPLC-MS-MS. In case of ADMA and SDMA n = 6 for WT and n = 7 for *Hnf4a* knockout mice, in case of BAIB n = 7 for both WT and *Hnf4a* knockout mice; error bars indicate SEM, Mann-Whitney test: ADMA *P = 0.0291; SDMA *P = 0.0231. Levels of BAIB in all wild type samples were below quantification limit of 0.2 μmol/L (N.D.–non detectable). **(c)** AGXT2 products levels in plasma of liver-specific *Hnf4a* knockout mice and wild type littermates. ADGV and SDGV concentrations were determined by HPLC-MS-MS. n = 7 for both WT and *Hnf4a* knockout mice. For SDGV culture-derived isotope labelled [2H6]-SDGV was used as the internal standard, therefore, only relative units (area ratios) are provided. Error bars indicate SEM, Mann-Whitney test: **P = 0.0014; ***P = 0.0006.

Next, we determined, whether liver-specific *Hnf4a* deficiency affects tissue AGXT2 activity and systemic levels of AGXT2-related metabolites. We observed an 85% decrease in tissue AGXT2 activity in the liver and no changes in AGXT2 activity in the kidneys of the liver-specific *Hnf4a* knockout mice (Fig. 6a). The plasma levels of the AGXT2 substrates ADMA and SDMA were increased by 18.3% and 23.3% (p < 0.05), while the plasma levels of the corresponding AGXT2 products ADGV and SDGV were decreased by 67.9% and 74.8%, respectively (p < 0.05) (Fig. 6b,c). Further, a dramatic increase in the plasma levels of the AGXT2 substrate BAIB was observed in *Hnf4a* knockout mice, whereas no BAIB was detected in the plasma of the wild-type littermates (Fig. 6b).

## Discussion

In this study we applied several complementary *in vitro* and *in vivo* approaches to demonstrate that HNF4α is the major regulator of *AGXT2* gene expression. First, *in silico* analysis predicted the presence of a functionally active HNF4α binding site in the mammalian *AGXT2* promoter. The role of this site during murine *Agxt2* promoter activation was demonstrated *in vitro* by mutational analysis and subsequent detection of the resulting decrease in promoter activity in luciferase reporter assays. Direct binding of HNF4α to the predicted consensus motif in the *Agxt2* promoter was confirmed by ChIP assay. The role of HNF4α in regulation of *Agxt2* expression in cultured hepatocytes was demonstrated by detection of a significant decrease in *Agxt2* mRNA levels after siRNA-mediated *Hnf4a* knockdown. In the *in vivo* part of our study we showed that inducible liver-specific *Hnf4a* knockout led to a dramatic downregulation of *AGXT2* expression and activity in the murine liver. Furthermore, deletion of *Hnf4a* in the murine liver led to an elevation of plasma levels of the AGXT2 substrates ADMA, SDMA and BAIB and a decrease in plasma levels of the AGXT2 products ADGV and SDGV, thus replicating the previously reported biochemical phenotypes of patients and experimental animals with reduced or absent AGXT2 activity.

AGXT2 is a mitochondrial pyridoxal phosphate-dependent amino transferase. It is encoded by a single gene located on the chromosome 5 in humans and on the chromosome 15 in mice. We found that the sequence −211 to +1 relative to the translation start site of murine *Agxt2* was sufficient for driving *Agxt2* promoter activity in the Agxt2 promoter/luciferase reporter assay (Fig. 2b). This region is 66% homologous between mice and humans with the longest conserved sequence containing the HNF4α consensus binding site. Our study demonstrated the essential role of this HNF4α binding site in regulation of *Agxt2* expression in the murine liver. Presence of a homologous HNF4α binding site in the 5′ UTR of human AGXT2^21^,^39^ suggests that HNF4α also regulates hepatic *AGXT2* expression in humans. This is consistent with high RNA polymerase II (Pol2) occupancy of the AGXT2 promoter region in both mouse and human liver (Fig. 1c). Interestingly, both *HNF4A* and *AGXT2* are coexpressed in the renal proximal tubules, raising the possibility that HNF4α might also be involved in regulation of *AGXT2* expression in the kidneys (GEO GDS3397^40^). This possibility is also supported by high Pol2 occupancy of the predicted AGXT2 core promoter region in the kidneys (Fig. 1c).

While outside the scope of this study, our analyses indicate that additional factors may be involved in transcriptional regulation of *AGXT2*. Indeed, while levels of *AGXT2* and *HNF4a* mRNAs generally correlate in both mouse and human tissues, small intestine represents a peculiar exception, expressing only *HNF4a*, but not *AGXT2*^41^. This is likely explained by either tissue-specific mechanism for negative regulation of *Agxt2* expression, such as DNA methylation, or local chromatin modifications that may prevent HNF4α binding or activity. Alternatively, additional cofactor(s), absent in small intestine, may be required for efficient HNF4α-dependent activation of *Agxt2*. Another possibility is that only certain isoforms of HNF4α are involved in regulation of *Agxt2* expression. Indeed, *HNF4a* gene contains two promoters, one with kidney and liver expression (P1) and one with pancreatic-specific expression (P2), and has 13 exons, which encode twelve distinct isoforms as the result of alternate promoter usage and differential splicing. The expression profile of these isoforms varies with development, differentiation, and tissue origin^42^,^43^,^44^. The P1-driven isoforms are known to have stronger transcriptional potentials, compared to the P2-originated ones, due to presence of AF-1 activation function domain in their structure^45^. Moreover, the downstream targets of HNF4α include the genes, expression of which is strictly or mainly dependent on the presence of a functional AF-1 domain in the HNF4α protein (*CAR, apoAIV, apoCII*), and the genes, expression of which is independent of this motif, although dependent upon HNF4α (*OTC* and *apoAII*)^46^. The experimental approaches, utilized in the current manuscript, were targeting all isoforms of HNF4α, so determination of the role of the specific isoforms of HNF4α in regulation of *Agxt2* expression in different tissues lies beyond the scope of the current work and will be examined in our future studies.

Interestingly, we did not observe complete loss of *Agxt2* expression in the liver of the liver-specific *Hnf4a* knockout mice (Fig. 5b) suggesting that the basal *Agxt2* transcription is independent of HNF4α. This is consistent with the proposed mechanism of HNF4α action, wherein it interacts with and augments transcriptional activation by the Mediator complex^47^. Additionally, HNF4α has been demonstrated to facilitate recruitment of histone acetyltransferase coactivators and to increase promoter accessibility for transcription factors via interaction with ATP-dependent chromatin remodelers^47^.

An almost 90% decrease in hepatic *Agxt2* expression, which we detected in the liver-specific *Hnf4a* knockout mice in the current study, did not result in compensatory changes in expression of *Ddah1* or *Ddah2* genes in the liver or compensatory changes in *Agxt2* and *Ddah1* expression in the kidneys. The levels of *Ddah2* in the kidneys were slightly elevated (Fig. 5b) possibly suggesting presence of an inducible tissue-specific enhancer, which still remains to be identified. Potential trigger for upregulation of *Ddah2* expression in the kidney could be increased circulating levels of ADMA, as a part of a possible negative feedback loop. Interestingly, upregulation of *Ddah2* expression in the kidneys was not capable of compensating the elevation of systemic ADMA levels, which was observed in our experiment, suggesting insufficiency of the potential endogenous DDAH2-mediated compensatory mechanisms regulating systemic ADMA levels.

Downregulation of *Agxt2* expression in the liver led to elevation of systemic levels of both ADMA and SDMA, which mimicked the metabolic phenotype observed by Caplin and colleagues in mice with global *Agxt2* deficiency^14^. These findings emphasize the essential role of hepatic AGXT2 in regulation of plasma levels of endogenous methylarginines and argue against the initial hypothesis of Lee and colleagues that AGXT2 is primarily a renal enzyme^48^. The major contribution of hepatic AGXT2 to total AGXT2 activity, however, is consistent with the previous reports from our and other groups, which showed strong expression of AGXT2 in the liver^15^,^30^,^32^,^49^.

Hepatic AGXT2 deficiency in response to liver-specific *Hnf4a* knockout resulted in appearance of micromolar levels of BAIB in plasma, whereas plasma levels of BAIB in the wild type littermates remained below the detection limit. Hence, *Hnf4a* knockouts are mimicking the biochemical phenotype of the patients with hyper-β-aminoisobutyric aciduria caused by AGXT2 deficiency^50^. Our data suggest that hepatic AGXT2 not only plays a major role in metabolism of endogenous methylarginines, but is also essential for systemic BAIB homeostasis. It is possible, however, that dramatic elevation of systemic BAIB concentration in liver-specific *Hnf4a* knockout mice is due not only to deficiency of AGXT2, but also to alterations in expression of some other genes involved in BAIB metabolism or transport. One such gene may be solute carrier family 6 member 13 (*Slc6a13, Gat2*), a known transporter of BAIB that is associated with hyper-β-aminoisobutyric aciduria in humans^17^. Supporting this hypothesis, gene expression analyses suggest that this transporter is almost completely depleted in the liver of *Hnf4α* knockout mice^23^.

Our finding that even partial downregulation of HNF4α either in cultured hepatocytes after siRNA-mediated *Hnf4α* knockdown (Fig. 4b) or *in vivo* in heterozygous *Hnf4a* knockout mice (Fig. 5c) still leads to a statistically significant proportional decrease in *Agxt2* expression suggests that the effect of HNF4a on *Agxt2* transcription is dose-dependent, which makes our study relevant to the large number of patients with mild HNF4α deficiency. Indeed, while severe inborn HNF4α deficiency is rare and leads to maturity-onset diabetes of the young 1 (MODY1)^24^, mild HNF4α deficiency due to *HNF4A* polymorphisms in either intronic or promoter regions is common and is associated with increased risk of type 2 diabetes mellitus^25^,^51^, gestational diabetes mellitus^52^, metabolic syndrome, dyslipidemia^53^ and arterial hypertension^54^. For instance, the allele frequency for common *HNF4A* polymorphisms rs2144908, rs3818247 and rs1884614, which are associated with higher susceptibility to type 2 diabetes, is approximately 20% in healthy population and increases up to 29% in type 2 diabetes patients^55^,^56^.

The finding that HNF4α deficiency and subsequent downregulation of AGXT2 leads to an increase in plasma ADMA concentration might also be clinically relevant, as the epidemiological studies have shown that even modest elevation in plasma ADMA levels is associated with increased risk of cardiovascular events and mortality in people with cardiovascular disease^57^,^58^. Furthermore, the pathophysiological significance of even modest changes in plasma ADMA concentration has been demonstrated in experimental studies^59^. A potential explanation for this phenomenon is that ADMA accumulates in endothelial cells *in vivo* at the levels 5–10 folds higher than the levels of ADMA in plasma, so the magnitude of ADMA changes in plasma might only partially reflect the levels in the intracellular compartments^60^. What further supports the potential clinical relevance of our findings is that in the *in vivo* part of our study we downregulated HNF4α only in one tissue, i.e. in the liver, and this alone led to significant changes in plasma levels of dimethylarginines. We speculate that downregulation of HNF4α levels in multiple tissues would lead to even more profound changes in metabolism of ADMA and SDMA.

Changes in AGXT2 activity can affect several metabolites, such as endogenous methylarginines^13^, BAIB^31^,^61^, NO^13^ and lipids^9^,^62^, which are all known to be dysregulated in diabetes. In addition, studies in animal models have demonstrated that AGXT2 deficiency leads to hypertension^14^, a classic feature of the metabolic syndrome. Furthermore, *AGXT2* polymorphisms have been linked to increased carotid intima-media thickness^63^, and, in diabetic patients, to increased risk of coronary artery disease^64^. These observations raise the possibility that HNF4α-related diabetes may represent a metabolically-distinct subset of diabetes characterized by impairment in AGXT2 activity and a higher risk for development of cardiovascular complications due to the resulting metabolic abnormalities. Published data regarding the association between diabetes and circulating levels of methylarginines such as ADMA are contradictory^65^,^66^, perhaps because HNF4α-related diabetes represents only a subset of patients. Testing this hypothesis by assessing ADMA- and BAIB-related metabolic changes as well as the spectrum and prevalence of cardiovascular complications in the diabetic patients with *HNF4A* polymorphisms may lead to development of novel targeted therapeutic approaches for this subgroup of patients.

## Additional Information

**How to cite this article**: Burdin, D. V. *et al*. Diabetes-linked transcription factor HNF4α regulates metabolism of endogenous methylarginines and β-aminoisobutyric acid by controlling expression of alanine-glyoxylate aminotransferase 2. *Sci. Rep.* **6**, 35503; doi: 10.1038/srep35503 (2016).

## Supplementary Material

Supplementary Information
