# Supplementary material for: Diabetes-linked transcription factor HNF4α regulates metabolism of endogenous methylarginines and β-aminoisobutyric acid by controlling expression of alanine-glyoxylate aminotransferase 2
Source: Sci Rep. 2016 Oct 18;6:35503. doi: 10.1038/srep35503 (PMC5067591; doi:10.1038/srep35503)
Supplement: Supplementary Information [file srep35503-s1.pdf]

Diabetes-linked transcription factor HNF4α regulates metabolism of endogenous methylarginines and β-aminoisobutyric acid by controlling expression of alanine-glyoxylate aminotransferase 2

Dmitry V Burdin[1]§, Alexey A Kolobov[2]§, Chad Brocker[3], Alexey A Soshnev[4] , Nikolay Samusik[5], Anton V Demyanov[6], Silke Brilloff[7], Natalia Jarzebska[7], Jens Martens-Lobenhoffer[8], Maren Mieth[9], Renke Maas[9], Stefan R Bornstein[10], Stefanie M Bode-Böger[8], Frank Gonzalez[3], Norbert Weiss[7], Roman N Rodionov[7]*

[1] Department of Physiology, Saint-Petersburg State University, 199034 Saint-Petersburg, Russia

[2] Department of Biochemistry, Saint-Petersburg State University, 199034 Saint-Petersburg, Russia

[3] National Cancer Institute, NIH, Bethesda, MD, 20892, USA

[4] The Rockefeller University, New York, NY, 10065, USA

[5] Stanford University School of Medicine, Stanford, CA, 94305, USA

[6] Institute of Highly Pure Biopreparations, 197110 Saint-Petersburg, Russia

[7] University Center for Vascular Medicine, Technische Universität Dresden, 01307 Dresden, Germany

[8] Institute of Clinical Pharmacology, Otto-von-Guericke University, 39120 Magdeburg, Germany

[9] Institute of Experimental and Clinical Pharmacology and Toxicology, Friedrich-Alexander-University Erlangen-Nürnberg (FAU), 91054 Erlangen, Germany

[10] Department of Internal Medicine III, University Hospital Carl Gustav Carus, Technische Universität Dresden, 01307 Dresden, Germany

§ These authors contributed equally to this work

* To whom correspondence should be addressed **:** Roman N. Rodionov, MD, PhD, University Center for Vascular Medicine, University Hospital "Carl Gustav Carus", Technische Universität Dresden, Fetscherstraße 42, 01307 Dresden, Germany, Phone: +49-351-458-3659, Fax: +49-351-458-4359, E-mail: Roman.Rodionov@uniklinikum-dresden.de

Name	Sequence (5'-3')	Source
Cloning		
End F	GCTCTCGAGGCCTCCATTTTTCTCATTATTTAAGGT	
End R	ACAAAGCTTCTCTTCCCAGGCAGAAAGAA	
Mut 1 F	TCTTCTAATATCAAAGTCGAGTCCAGTCAGTGAGC	
Mut 1 R	GCTCACTGACTGGACTCGACTTTGATATTAGAAGA	
Mut 2 F	TCTTCTAATATCTCAGTCGAGTCCAGTCAGTGAGC	
Mut 2 R	GCTCACTGACTGGACTCGACTGAGATATTAGAAGA	
Mut 3 F	TCTTCTAATATAGCCTTCGAGTCCAGTCAGTGAGC	
Mut 3 R	GCTCACTGACTGGACTCGAAGGCTATATTAGAAGA	
Mut 4 F	TCTTCTAATCAAGCCTCAGAGTCCAGTCAGTGAGC	
Mut 4 R	GCTCACTGACTGGACTCTGAGGCTTGATTAGAAGA	
qPCR for mRNA quantification		
Agxt2 F	sequence not enclosed	Qiagen QT01557682
Agxt2 R	sequence not enclosed	Qiagen QT01557682
Hnf4a F	AGAAGATTGCCAACATCAC	
Hnf4a R	GGTCATCCAGAAGGAGTT	
Hnf4a F2	AGAAGATTGCCAACATCAC	
Hnf4a R2	GGTCATCCAGAAGGAGTT	
Ddah1 F	CTACGCAGTCTCTACAGT	
Ddah1 R	TCATAACGATGGTCACTCA	
Ddah2 F	AAAGCAGTCAGGGCAATG	
Ddah2 R	CCAGGACGCAGAAAGAGA	
HPRT F	CTTTGCTGACCTGCTGGATTAC	
HPRT R	ATCCAACACTTCGAGAGGTCC	
Gclc F	CTACCACGCAGTCAAGGACC	1
Gclc R	CCTCCATTCAGTAACAACTGGAC	1
RpL13 F	ATCCCTCCACCCTATGACAA	
RpL13 R	GCCCCAGGTAAGCAAACTT	
qPCR for ChIP analyses		
Exp 1 F	AAGTCCATTTATAGTCAGCAGTCT	
Exp 1 R	CTGTGGAAGCAGGTCAGC	
Exp 2 F	CCCAAGTCCATTTATAGTCA	
Exp 2 R	TCCAGGCTAGAGACATC	
Neg 1 F	CGTGGTTGTTACTAAGTGTGTTCA	
Neg 1 R	TGGTATGTGCTCATTCTTGTCTCA	
Neg 2 F	CATCTGTCTTGCTCAGTTCTT	
Neg 2 R	GCTGTTGTGTAGTGCTCTC	
Pos 1 F	CAAGGCTGAAGTCCAAAG	2
Pos 1 R	CTGCTCTGTTTACATTGGA	2

Supplementary Table S1. Sequences of PCR and cloning primers used in work. Primers, for which the source is not specified, were self-designed.

References:

1. Patwardhan, R. S., Checker, R., Sharma, D., Sandur, S. K. & Sainis, K. B. Involvement of ERK-Nrf-2 signaling in ionizing radiation induced cell death in normal and tumor cells. *PloS One* **8,** e65929 (2013).

2. Cozzolino, A. M. *et al.* TGFβ overrides HNF4α tumor suppressing activity through GSK3β inactivation: implication for hepatocellular carcinoma gene therapy. *J. Hepatol.* **58,** 65–72 (2013).
